# The Symptoms of Surgical Affections of the Kidneys

**Published:** 1892-04-02

**Authors:** 


### SURGERY.

### THE SYMPTOMS OF SURGICAL AFFECTIONS OF THE KIDNEYS.

It is becoming more and more difficult to draw a line of demarcation between the medical and surgical diseases of internal organs, and there is no doubt a gradually increasing tendency for the surgeon to claim as his many conditions which a short time ago belonged exclusively to the physician. The scalpel and drainage tube are now admitted to the sacred provinces of the liver and the kidney, the stomach and the lungs. It has even been suggested that all organic affections should be considered surgical unless they are known to be hopeless, in which case they should be handed over to the physician. This amicable contest is well illustrated in the kidney; but; without attempting to settle any moot points of this nature, we may take it for granted that all local inflammatory and obstructive conditions in the kidney and its pelvis, whether from tumours, calculi, injury, or other causes, may be considered as distinctly "surgical." Their symptoms, of course, vary immensely, and absolutely certain diagnosis is rare. Nevertheless, a great deal can be done to ascertain the nature of the lesion, and few men have done more to elucidate this matter than Professor Guyon. He divides the signs of kidney disease into two heads—(1) changes in the quantity or quality of the urine; and (2) more remote manifestations (*e.g.*, fever, pain, &c.). Dr. Henri Hartmann (in *Le Progrès Médical* for March 5th, 1892) gives a very good outline of some of the modern methods of diagnosing these affections. What follows is partly abstracted from this article.

Firstly, as regards the urine. In the early stages this is generally increased in amount, and at first clear, afterwards usually containing some deposit. The latter is not of the thick, glairy nature found in cystitis, and fermentation does not so easily occur, probably owing to the comparative absence of urea. Pus is, by itself, of little diagnostic use, but occasional purulent urine, with healthy and clear intervals, shows (1) that probably there is renal and not vesical suppuration, and (2) that the disease is unilateral.

Blood from the kidney is well mixed with the urine, giving the "smoky" tint; this may be renal or vesical, but long blood casts, formed in the ureter, are characteristic. It is only when of considerable length that these are of importance, however. Short ones may come from the urethra. It is well known that sudden hæmaturia may come from almost any part of the urinary apparatus; if very profuse, it is more indicative of renal than vesical hæmorrhage. According to Guyon, quick and frequent alternations of bloody urine can only occur from the kidney.

If the hæmorrhage is from a calculus movement will be apt to excite it, and complete rest to stop it; this does not hold with morbid growths; in these cases the urine is usually quite (or almost) clear before and after the bleeding.

Amongst the indirect symptoms of these diseases are variations in the temperature and digestive troubles. A remittent type of fever, lasting for some considerable time, and marked by irregular exacerbations (perhaps with two or more *maxima* in the twenty-four hours) is highly suggestive of calculus in the kidney. The tongue is red, dry, and blackish towards the centre (*langue urinaire*), but much reliance must not be placed on this. The appetite is usually poor, and sometimes thirst is a marked symptom. Almost all or any of these general signs may be absent or undecided, and examination of the diseased organ by manipulation, puncture, or exploratory incision must be had resort to. Examination through the abdominal walls may be carried out in several ways, the principles being (1) to relax the muscular parietes, (2) to force down the kidney by inspiration, and (3) to carefully select the accessible regions.

Glénard's method of palpation is the same as that usually carried out in England. The patient lies on his back, and if the right side is to be examined the surgeon grasps the relaxed abdominal wall below the ribs with his left hand, the fingers pressing into the flank, whilst the right hand exerts firm pressure in front.

Israël (who claims that by his plan a normal kidney can be felt) advises that the patient should lie on the opposite side to the one diseased, with the legs flexed. The hands clasp the loin in front and behind, the fingers of the anterior hand pressing inwards about one inch below the junction of the ninth and tenth costal cartilages. Guyon gives minute but useful details as to his process. The bowels must be evacuated the evening before, and no food taken after this until the examination, to ensure empty stomach and intestines. The patient lies on his back with extended and relaxed limbs. The surgeon insinuates one hand behind the loins, so that the fingers may be thrust into the angle between the last ribs and the first lumbar vertebræ. The other hand is in front. By this method, a "ballottement" of the kidney may be effected, which is of great use, the organ being jerked forwards and felt impinging anteriorly. If the enlargement (supposing that there is a tumour to be diagnosed) is most marked in the "costo-vertebral" angle, behind, it is almost certain to be kidney. Some rather fanciful procedures have been recommended as aids to diagnosis in these cases, such as catheterisation of one ureter, &c.; but if no certainty can be arrived at by palpation and a study of the symptoms, an exploratory incision must be made, either in the loins or in front, the latter being probably the best, as both kidneys can be examined. If there is no doubt as to the side of the disease, and there is reason to think the organ is not enlarged, however, a lumbar incision is the easiest. Puncture with a fine, long trocar may ascertain the existence of fluid, possibly of calculus; but the chance of wounding other organs renders this a doubtful means of diagnosis.

It is satisfactory to know that during the last few years sufficient progress has been made in the diagnosis and treatment of these painful disorders to rescue many of them from the list of hopeless diseases.

